# Curriculum evolution and documentary evidence of competency alignment in a hospital-embedded undergraduate health services and management programme in China: a longitudinal case study

**DOI:** 10.3389/fpubh.2026.1956968

**Published:** 2026-09-24

**Authors:** Jin Tian, Liang Dong, Yuchang Li, Lejiao An, Liang Sun, Zengren Zhao

**Affiliations:** 1 Hospital Management Innovation Research Center, The First Hospital of Hebei Medical University, Shijiazhuang, Hebei, China; 2 Department of Healthcare-Associated Infection Control, The First Hospital of Hebei Medical University, Shijiazhuang, Hebei, China; 3 Office of the President, The First Hospital of Hebei Medical University, Shijiazhuang, Hebei, China

**Keywords:** competency alignment, curriculum governance, curriculum mapping, health services and management, hospital-embedded education, practice education

## Abstract

**Background:**

Undergraduate Health Services and Management (HSM) programmes are expected to integrate management, medicine, public health, digital capability and workplace learning. Hospital-embedded delivery may provide access to authentic organizational settings and interdisciplinary expertise, but distributed teaching can fragment curriculum ownership, competency progression and assessment. This study examined longitudinal evolution and documentary evidence of competency alignment in a hospital-embedded undergraduate HSM programme in China.

**Methods:**

A longitudinal document analysis and competency-oriented curriculum-mapping case study was conducted from programme approval in 2020 to first-cohort graduation in 2026. Of 18 unique materials assessed, 15 programme-specific documents formed the empirical corpus, including original and revised training plans, course and teaching-unit records, faculty-development materials, internship documents, quality-assurance instruments and aggregate graduate-destination data. Documentary evidence was assessed using 33 indicators across seven domains and traced through five analytical stages: stated, curricularised, practiced, assessed and fed back. Each indicator was rated as explicitly represented, partially represented or not identified. A second reviewer independently rescored all indicators.

**Results:**

Graduation requirements increased from 165 to 184.5 credits, required courses from 47 to 55, and five elective courses were listed in the revised plan. Management was positioned more clearly as a principal discipline, while biomedical, digital-health and artificial-intelligence content expanded. The 40-week internship covered hospital, public-health, community and industry settings and specified activity requirements, but no programme-wide cross-placement competency rubric was identified. Of 33 indicators, 11 (33.3%) were explicitly represented, 21 (63.6%) were partially represented and one (3.0%) lacked identifiable documentary evidence. Practice education showed the strongest documentary representation, whereas longitudinal digital-health competency development, programme-level assessment and completed feedback cycles were less fully documented. Reviewers agreed on 31 of 33 ratings (93.9%), with a quadratic-weighted Cohen's kappa of 0.893.

**Conclusions:**

Hospital embedding provided documented access to interdisciplinary teaching resources and diverse workplace-learning settings, but evidence of coherent competency progression, comparable assessment across settings, applied digital-health learning and completed feedback cycles remained limited. Curriculum ownership, longitudinal competency mapping, common assessment mechanisms and systematic feedback may help strengthen programme coherence. We propose a provisional, case-derived resource-to-capability conversion framework for further investigation in distributed professional programmes.

## 1 Introduction

Health systems increasingly require professionals who can connect population health needs with the organization, delivery and improvement of healthcare services. Undergraduate Health Services and Management (HSM) education responds to this need by integrating management, medicine, public health, digital capability and health-service practice. This interdisciplinary breadth, however, also creates a central curriculum challenge. The inclusion of content from several disciplines does not necessarily produce coherent professional competence. Competency-based education therefore requires programmes to move beyond lists of courses and specify how intended graduate capabilities are developed through learning activities, workplace experiences and assessment (1, 2).

Curriculum mapping provides a systematic means of examining this alignment. By linking intended outcomes with courses, learning activities and assessment, it can identify gaps, duplication and weak progression within a programme (3–5). Neumark et al. extended this approach by distinguishing whether public-health competencies were addressed, how extensively they were covered and whether students had opportunities to apply them (6). Their findings showed that the presence of a competency in one or more courses did not necessarily indicate sufficient applied learning. Programmatic assessment makes a related distinction. Competence should not be inferred from an isolated examination or the completion of prescribed activities, but should be supported by multiple and connected sources of evidence collected across a programme (7, 8). Curriculum evaluation should therefore examine not only whether intended capabilities are formally stated, but also whether they are incorporated into curriculum provision, practiced in authentic settings, assessed against defined standards and used to inform programme improvement.

Maintaining such alignment becomes more difficult when teaching is distributed across academic units and workplace settings. Distributed delivery can provide access to specialized teachers, healthcare managers and authentic organizational environments, but it may also disperse curriculum ownership and create variation in teaching, supervision and assessment. A scoping review found that educational comparability across distributed settings depended on common learning objectives, consistent assessment arrangements, clear governance, communication across sites, faculty development and monitoring of student outcomes (9). The development of an undergraduate Health Information Management programme in the Philippines similarly showed that competency definition and course design required support from institutional ownership, faculty capacity, infrastructure and external partnerships (10). These findings indicate that access to educational resources does not automatically create a coherent programme. Distributed contributions must also be coordinated through programme-level governance, longitudinal curriculum planning and comparable assessment arrangements.

In China, HSM was added to the national undergraduate programme catalog in 2015, and the first institutions began enrolling students in 2016 (11). Its subsequent development has been supported by national policies promoting health-management workforce development and interdisciplinary health education (12). Nevertheless, Chinese studies have reported recurring challenges involving unclear disciplinary positioning, imbalanced faculty resources, heterogeneous curriculum structures and insufficient practice education (13, 14). Curriculum mapping has recently been applied to clarify the disciplinary composition and sequencing of HSM education (15). Existing studies, however, have mainly described training objectives, curriculum structures or proposed reforms at a single point in time. Limited evidence is available on how a newly established programme develops from initial approval through curriculum revision, distributed course delivery, multi-setting workplace learning and the graduation of its first cohort. Evidence is particularly limited for programmes coordinated through affiliated hospitals, where access to healthcare expertise and authentic practice settings raises questions about how curriculum ownership, competency progression, assessment and quality improvement are coordinated across distributed teaching settings.

This study examined how a hospital-embedded undergraduate HSM programme at a Chinese medical university developed from its approval in 2020 to the graduation of its first cohort in 2026, with particular attention to documentary evidence of competency alignment. Qualitative document analysis and competency-oriented curriculum mapping were used to examine changes in programme positioning and curriculum structure, the representation of intended capabilities across the stages of being stated, curricularised, practiced, assessed and fed back, and gaps in the documentary record relating to governance, assessment and quality assurance. The study did not directly assess student competence, implementation fidelity or programme effectiveness. It also considered how hospital-based expertise and practice opportunities were organized at programme level to support curriculum development.

## 2 Materials and methods

### 2.1 Study design and setting

This longitudinal qualitative case study combined document analysis with competency-oriented curriculum mapping to examine the development of a newly established undergraduate HSM programme at a medical university in China. The study followed the programme from its initial design and curriculum revision to distributed teaching, workplace learning and the graduation of the first cohort in 2026.

The programme was approved in 2020, did not enroll students in 2021 and admitted its first cohort in 2022. It was coordinated through an affiliated teaching hospital rather than a conventional school of management, with teaching and practice responsibilities distributed across university teaching units, affiliated hospitals, external academics, hospital managers and practice organizations. In this study, hospital-embedded refers to an organizational arrangement in which programme coordination and a substantial proportion of teaching and practice resources were located within, or mobilized through, an affiliated teaching hospital.

### 2.2 Document corpus and data extraction

Programme-specific documents available up to 20 June 2026 were identified from curriculum-development, teaching-management and internship records and cataloged before eligibility assessment. A document was defined as one identifiable file or formally issued version, with revised versions treated separately when they represented distinct stages of programme development. Nineteen records were cataloged. After removal of one duplicate copy of the internship arrangement, 18 unique records remained for eligibility assessment. Fifteen programme-specific documents formed the empirical corpus, including the original and revised undergraduate training plans; required-course, elective-course and course-to-programme mapping materials; documents on teaching-unit structures and staff responsibilities; a faculty-development plan; an internal programme-reform report; the first-cohort internship schedule and handbook; teaching-quality monitoring instruments; and an aggregate summary of first-cohort graduate destinations. Documents were included when they were specific to the undergraduate HSM programme and contained information relevant to programme objectives, curriculum provision, teaching responsibility, faculty development, practice education, assessment, quality assurance or graduate transitions.

Three unique records were not coded as empirical evidence. A proposal concerning the later development of a graduate discipline in hospital management, dated 28 July 2026, was reviewed after closure of the empirical corpus and retained only as contextual material. A document on the digital upgrading of a vocational programme at another institution was used as an external sensitizing reference, while an undergraduate HSM curriculum from another university was excluded because it concerned a different institution and organizational context. The complete document inventory, eligibility decisions and analytical roles are presented in Supplementary Table S1.

A standardized extraction matrix recorded each document's title, type, date or version, issuing or responsible unit, analytical role, limitations, source identifier and location within the document. Extracted information included graduation credits, course numbers and categories, disciplinary positioning, teaching and faculty arrangements, internship settings and duration, required practice activities, supervision and recording arrangements, assessment outputs, quality-assurance mechanisms and graduate-destination categories. The indicator was the primary unit of coding, and each rating could be supported by one or more traceable extracts from one or more documents. Planned or proposed reforms were distinguished from documented implementation. Blank evaluation, inspection or monitoring templates were treated as evidence that an instrument existed, but not as evidence that it had been used, that findings had informed programme decisions or that a quality-improvement cycle had been completed. Inconsistent figures, unavailable attachments and unclear relationships between document versions were retained as evidence limitations rather than resolved through assumption.

All empirical source documents were in Chinese and were analyzed in the original language. English terms and extracts reported in the manuscript were translated by the first author and checked against the original wording during manuscript preparation.

### 2.3 Analytical framework and coding

The analysis drew on established curriculum-mapping principles (4–6) and a previously developed HSM programme-evaluation framework. The source framework comprised 33 terminal indicators developed through a modified Delphi process and analytic hierarchy process. The same indicator structure was retained for the present study, but the operational definitions were adapted to specify the forms of evidence that could be established from programme documents. The Delphi and analytic hierarchy process procedures were not repeated, and the original indicator weights were not used.

Indicators that originally referred to student, teacher, graduate or employer perceptions or outcomes were not inferred from institutional documents. Coding was limited to programme structures and processes that could be traced in the documentary corpus, including curriculum provision, teaching responsibilities, practice arrangements, assessment mechanisms and quality-assurance processes. The 33 indicators were organized into seven domains: programme objectives and disciplinary positioning; curriculum architecture and progression; distributed governance and faculty capacity; practice education and cross-setting comparability; assessment architecture; digital-health competency maturity; and quality assurance and feedback loops. Operational definitions, documentary evidence, source locations and ratings for all indicators are presented in Supplementary Table S2.

Each indicator was rated on a three-category documentary-evidence scale. A score of 0 indicated that no relevant evidence was identified in the empirical corpus. A score of 1 indicated partial, planned, inconsistent or otherwise incomplete documentary representation of the indicator. A score of 2 indicated that the elements specified in the operational definition were explicitly represented in the available documents. Ratings therefore reflected the completeness of documentary representation rather than educational quality, implementation fidelity or student competence. Documentary absence was likewise interpreted as absence of evidence in the available corpus, rather than evidence that an activity had not occurred.

Evidence was also traced across five stages: stated, curricularised, practiced, assessed and fed back. Stated referred to explicit recognition in programme objectives, policies or other formal documents. Curricularised indicated translation into a course, placement, learning requirement, responsibility structure or formal instrument. Practiced indicated documentary evidence of a defined opportunity or activity through which the relevant capability or programme mechanism could be enacted. Assessed referred to a documented assessment, performance record, criterion, rubric, monitoring procedure or decision mechanism relevant to the indicator. Fed back required documentary evidence that assessment or monitoring findings had subsequently informed a programme decision, curriculum revision or improvement action.

The overall 0–2 rating and the five-stage profile served different analytical purposes. The overall rating reflected how fully the operational definition of an indicator was represented in the documentary corpus, whereas the stage profile was used to identify where documentary evidence weakened between formal intention, curriculum provision, practice, assessment and feedback. A “Yes” at a particular stage indicated that relevant documentary evidence was identified at that stage; it did not by itself establish the quality, consistency or effectiveness of implementation. Stages that were not conceptually applicable to an indicator were recorded as not applicable. The complete five-stage matrix is presented in Supplementary Table S3.

### 2.4 Coding procedures and researcher positionality

The first author conducted the initial document review, data extraction and indicator-level coding and had been involved in programme coordination, curriculum development and teaching administration during the study period. This insider position provided useful knowledge of document provenance, version changes and organizational context, but it also meant that personal knowledge of the programme had to be kept separate from evidence contained in the documentary corpus.

Coding decisions were based on traceable documentary evidence. The first author's contextual knowledge was used to clarify the origin, timing and relationship of documents, but was not treated as evidence that a planned activity had been implemented or that a programme feature had been effective. Where contextual knowledge suggested an interpretation that was not supported by the documentary record, it was not used to fill documentary gaps or strengthen an indicator rating. Missing, inconsistent and unfavorable evidence was retained in the coding record, and ambiguous cases were resolved by returning to the source documents rather than by relying on recollection or programme experience.

A second reviewer, a senior academic in social medicine and health services research with no role in programme design, document preparation or routine programme operation, independently rescored all 33 indicators. The reviewer received the prespecified indicator definitions, scoring criteria, source identifiers and extracted evidence, but did not have access to the first reviewer's ratings or coding rationales. Original documents could be consulted when the extracted evidence was insufficient or when the context of a record required verification.

Inter-rater agreement was assessed after the independent review and before consensus discussion using percentage agreement and Cohen's kappa statistics.

Disagreements were resolved by re-examining the relevant source material against the prespecified criteria. Where the available documents did not support the stronger interpretation, the more conservative rating was retained. Final consensus ratings were used in the subsequent analyses.

### 2.5 Data analysis

Descriptive analysis compared the original and revised programme arrangements, including graduation credits, course numbers and categories, disciplinary positioning and internship time allocation. Final documentary-evidence ratings were summarized by indicator and domain using counts and percentages, with both numerators and denominators reported because the domains contained different numbers of indicators. The 33 indicators represented distinct aspects of programme design and implementation rather than a single underlying construct; accordingly, no internal-consistency coefficient, weighted aggregate score or composite programme-quality score was calculated.

Framework-guided qualitative content analysis examined relationships among programme objectives, curriculum provision, governance and faculty arrangements, practice opportunities, assessment mechanisms and feedback processes.

The five-stage matrix was used to trace documentary evidence from formal intention through curriculum provision, practice, assessment and feedback and to identify where this documentary chain became incomplete. Practice arrangements were also compared across hospital, public-health, community and industry settings. Diversity of placement settings was not taken as evidence of comparability unless common learning expectations, supervision arrangements, recording requirements or assessment standards were documented. Stage-level findings were interpreted as patterns in the documentary record rather than as measures of programme maturity or educational effectiveness.

Graduate-destination data were analyzed separately as early transition information available at graduation. The source categories were retained as reported and were not used to infer employment stability, job–programme alignment or workplace competence.

## 3 Results

### 3.1 Document corpus and programme development

Fifteen programme-specific documents formed the empirical corpus, covering initial programme design, curriculum revision, distributed teaching arrangements, the first-cohort internship, quality-monitoring instruments and early graduate-transition data. The programme was approved in 2020, admitted its first cohort in 2022 and reached first-cohort graduation in 2026. Figure 1 summarizes the main stages of programme development and the documentary evidence available at each stage.

**Figure 1:**
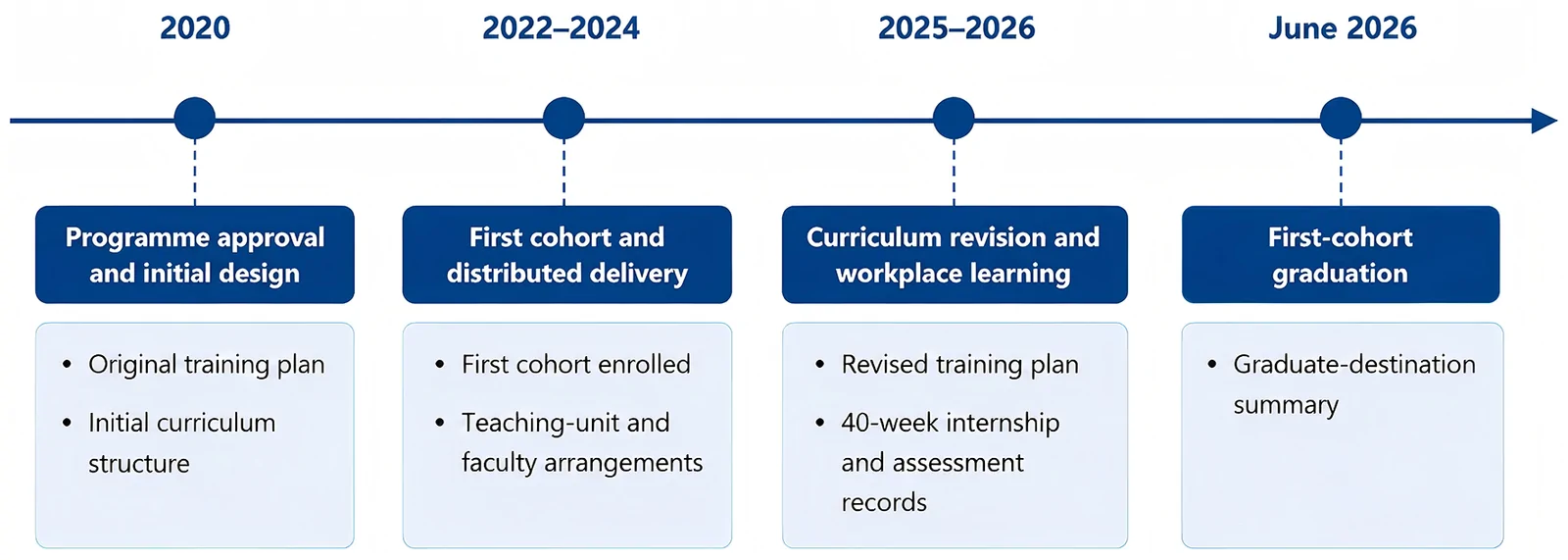
Programme development and principal documentary evidence, 2020–2026. The timeline summarizes the main programme milestones and the principal categories of documentary evidence available at each stage. The complete document inventory is provided in Supplementary Table S1.

### 3.2 Evolution of programme positioning and curriculum architecture

The original and revised training plans differed in curriculum scale, disciplinary positioning and the visibility of digital-health content (Table 1). Graduation requirements increased from 165 to 184.5 credits, and the number of required courses increased from 47 to 55. Five elective courses were listed in the revised plan. Because the original elective provision was documented in a separate attachment whose relationship to the revised plan could not be confirmed, changes in total elective provision were not quantified.

**Table 1:** Comparison of the original and revised undergraduate training plans.

Curriculum feature	Original plan	Revised plan	Documented difference
Graduation credits	165	184.5	+19.5 credits
Required courses	47	55	+8 required courses
Elective provision	Original elective attachment available; comparability with revised list uncertain	Five electives listed	Direct comparison not possible
Principal disciplinary positioning	Management not consistently identified as the principal discipline	Management explicitly identified as a main discipline	Clearer management orientation
Biomedical and health-science content	Present	Expanded	Broader documented coverage
Digital health and artificial intelligence	Limited or general references	More explicit in objectives and course descriptions	Greater documentary visibility
Interdisciplinary progression	No clearly documented staged integration	Broader interdisciplinary intentions, but no explicit progression sequence	Expansion clearer than longitudinal integration

Only differences supported by the available documentary corpus are reported; uncertain relationships between document versions were not inferred.

The original plan combined general education, biomedical sciences, public health and management-related content, but management was not consistently identified as the programme's principal disciplinary foundation. In the revised plan, management was explicitly identified as a main discipline and was given greater prominence in the programme objectives and curriculum structure. The revised plan also included more biomedical and health-science content within the higher overall curriculum load. Digital health, health information and artificial intelligence were stated more explicitly in the revised programme objectives and course descriptions.

The revised documents therefore showed a clearer formal positioning of management and broader interdisciplinary curriculum provision. The documentary record did not, however, show a clearly defined four-year sequence through which management, biomedical and public-health knowledge were progressively introduced, integrated, applied and assessed. Curriculum expansion and disciplinary repositioning were more clearly documented than longitudinal integration and progression.

### 3.3 Distributed governance and faculty capacity

The programme used a distributed organizational model in which teaching responsibilities were allocated across university teaching units, affiliated hospitals, external academics and healthcare practitioners. Formal documents assigned individual courses and teaching functions to responsible units and specified staff responsibilities for syllabus development, teaching plans, teaching materials and assessment preparation. The allocated teaching responsibilities spanned medicine, public health, statistics, management and healthcare practice.

Programme-level coordination was less clearly established. Although the documents described multi-unit teaching, collective lesson preparation and proposed roles for hospital managers and external practice experts, they did not show a sustained mechanism for coordinating curriculum sequencing, assessment and review across teaching units. Records of continuing participation by hospital-based and external contributors were limited, and the faculty-development plan identified shortages of staff with directly relevant HSM backgrounds and limited dedicated staffing. Overall, course-level responsibility was more clearly defined than collective responsibility for the curriculum as a whole. Of the five indicators in this domain, one was explicitly represented and four were partially represented.

### 3.4 Practice education and cross-setting comparability

The first-cohort internship was organized over 40 weeks across hospital health-management centers, clinical or functional departments, a provincial center for disease control and prevention, a community health-service center and university–industry placements (Table 2).

**Table 2:** Structure and common requirements of the 40-week internship.

Placement setting	Weeks	Proportion, %	Documented focus
Hospital health-management centers	8	20	Health assessment, health-management records, health education and follow-up
Clinical or functional departments	16	40	Clinical-service processes, departmental operations and administrative functions
Provincial center for disease control and prevention	4	10	Disease prevention, surveillance and public-health practice
Community health-service center	4	10	Primary care, community health management and chronic-disease follow-up
University–industry placement	8	20	Management-related and industry-based practice
**Total**	**40**	**100**	Five placement settings
Common minimum activities across the internship	–	–	At least 200 health-management records, 30 health-education activities and 100 chronic-disease follow-ups
Required records and assessment outputs	–	–	Internship records, departmental evaluations, a learning reflection and an internship report

The quantitative activity requirements applied across the full internship rather than separately to each placement. Bold values indicate the total internship duration (40 weeks) and the corresponding overall proportion (100%).

The internship documents specified common minimum requirements across the placement period, including at least 200 health-management records, 30 health-education activities and 100 chronic-disease follow-ups, together with internship records, departmental evaluations, a learning reflection and an internship report. The handbook also set out setting-specific learning tasks for different practice environments.

The practice model was more standardized in placement structure and activity requirements than in the assessment of performance across settings. Although supervision, recording and sign-off arrangements were formally specified, the documents did not provide a programme-wide rubric with common performance anchors or weighting across placement sites. Assessment could be organized according to local circumstances, limiting the comparability of performance evidence between settings. Of the five indicators in this domain, four were explicitly represented and one was partially represented.

### 3.5 Assessment, digital-health capability and quality assurance

Assessment was documented at both course and placement levels. Course assessment included formative and summative components such as classroom tests, assignments, examinations and skills assessments, while internship assessment drew on activity records, ongoing performance, departmental evaluation, reflection and a final report.

These sources provided multiple forms of assessment evidence, but they were not brought together within a programme-level framework linking graduate capabilities to evidence across courses and placements. Common behavioral standards for management competence and cross-setting performance were also not consistently specified.

Digital health, health information and artificial intelligence became more visible in the revised programme objectives and curriculum. Relevant provision included formal courses and a smart-hospital placement, but the documentary record showed limited progression from stated objectives to defined applied tasks and assessment. No dedicated assessment of digital-health or artificial-intelligence capability was identified. None of the four indicators in this domain was explicitly represented; three were partially represented and one had no identifiable documentary evidence.

Quality-assurance documents included professional-theory course evaluation forms, mid-semester inspection forms and feedback instruments. These documents specified formal instruments for monitoring teaching, but evidence of a complete cycle from data collection and review to programme action and subsequent reassessment was limited. One of the five indicators in this domain was explicitly represented and four were partially represented.

### 3.6 Cross-domain documentary evidence and five-stage translation

The reviewers agreed on 31 of the 33 indicator ratings before consensus discussion, giving 93.9% exact agreement. Unweighted Cohen's kappa was 0.879, linear-weighted kappa was 0.884 and quadratic-weighted kappa was 0.893. The two disagreements concerned indicators 1.2 and 3.5; both were assigned a final score of 1 after the source evidence was re-examined. Based on the final consensus ratings, 11 indicators (33.3%) were explicitly represented, 21 (63.6%) were partially represented and one (3.0%) had no identifiable documentary evidence.

The distribution differed across domains (Figure 2). Practice education and cross-setting comparability had four of five indicators explicitly represented, programme objectives and disciplinary positioning had two of four, and assessment architecture had two of five. Curriculum architecture and progression, distributed governance and faculty capacity, and quality assurance and feedback loops each had one of five indicators explicitly represented. None of the four digital-health indicators was explicitly represented.

**Figure 2:**
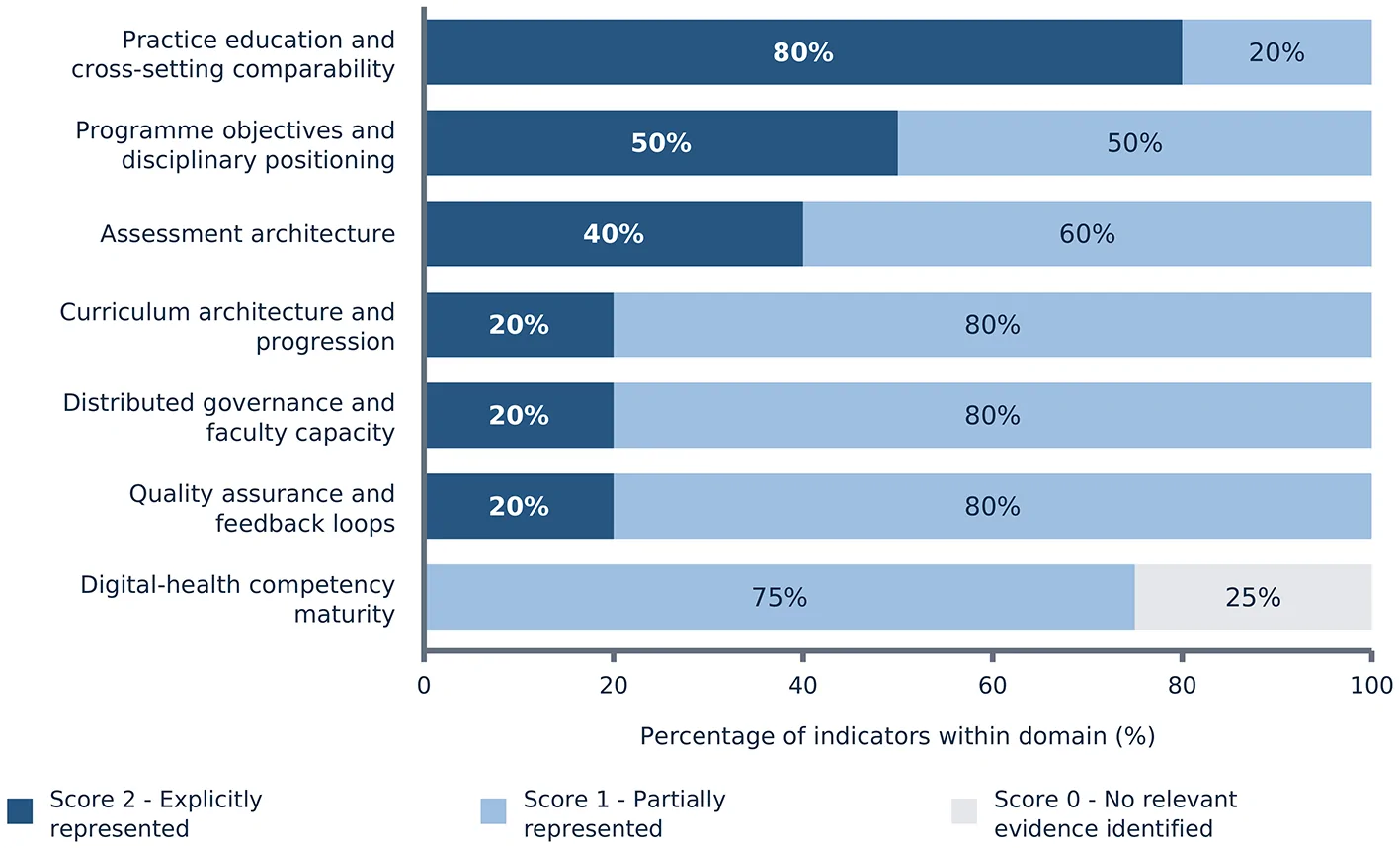
Distribution of documentary-evidence ratings across the seven domains. Bars show the within-domain proportions of indicators rated 2 (explicitly represented), 1 (partially represented), and 0 (no relevant evidence identified).

The five-stage analysis showed a clear difference between formal curriculum provision and later stages of translation. Evidence was identified for 18 of 19 applicable indicators (94.7%) at the stated stage and for 31 of 33 indicators (93.9%) at the curricularised stage. This decreased to 14 of 32 applicable indicators (43.8%) at the practiced stage and eight of 32 (25.0%) at the assessed stage; no indicator met the criterion for the fed-back stage (Figure 3). Practice-education indicators showed the greatest continuity into documented activity and assessment, whereas digital-health evidence was concentrated mainly at the stated and curricularised stages. Overall, formal intentions were more consistently translated into curriculum structures than into documented application, comparable assessment and programme-level feedback.

**Figure 3:**
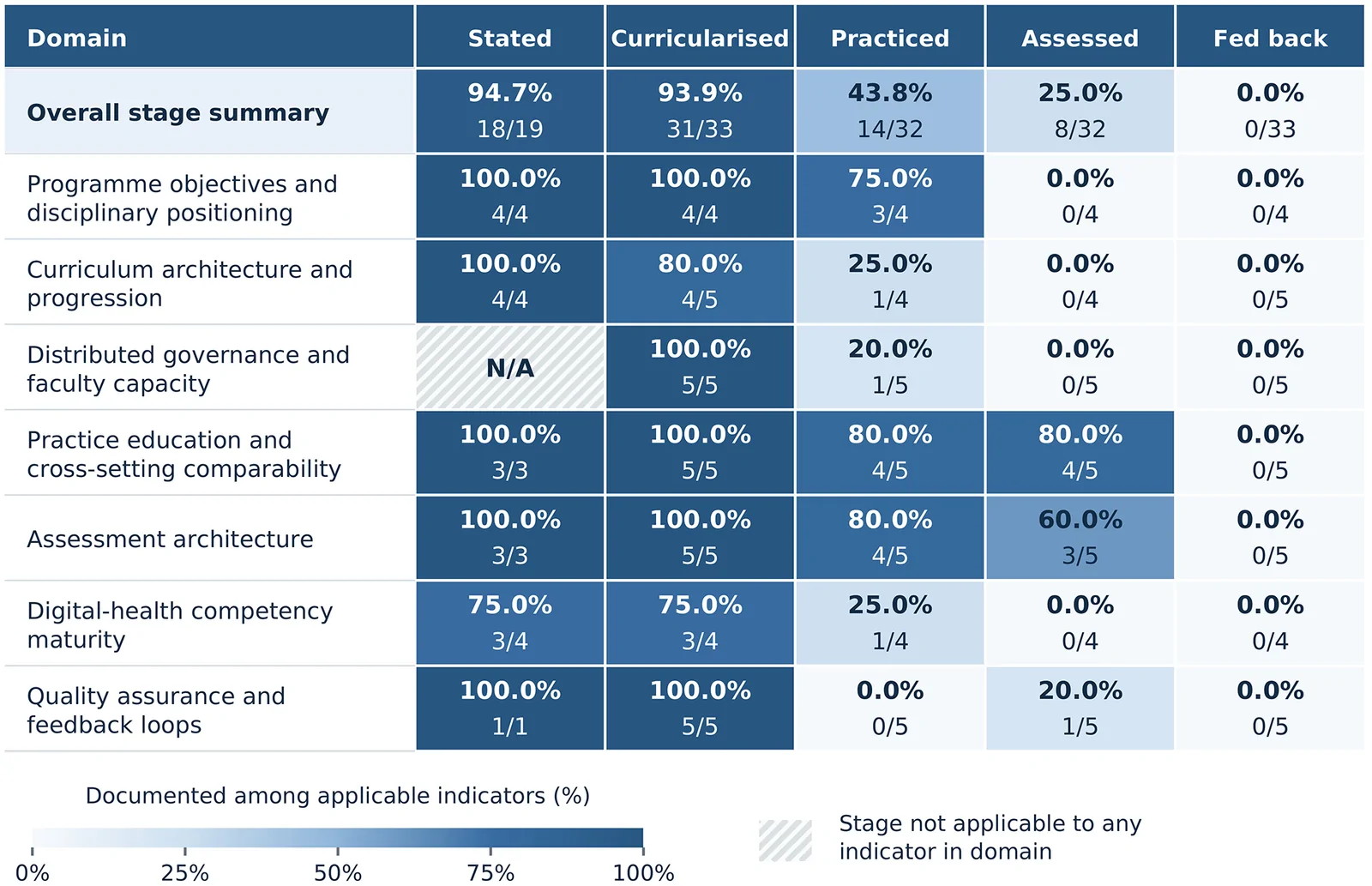
Domain-level documentary evidence across five curriculum-translation stages. Cells show the number and percentage of applicable indicators for which documentary evidence was identified at the stated, curricularised, practiced, assessed and fed-back stages. Hatched cells indicate that the stage was not applicable to any indicator within that domain. The overall stage summary is shown in the first row. Full indicator-level results are provided in Supplementary Table S3.

### 3.7 Early destinations of the first graduating cohort

The available graduate-transition record captured the destinations of all 28 students in the first graduating cohort at graduation in June 2026. Seven graduates (25.0%) progressed to further study: four through postgraduate entrance examinations, two through recommendation-based admission and one through overseas postgraduate study. Three (10.7%) entered civil-service or public-sector recruitment pathways, one (3.6%) entered a state-owned enterprise and 17 (60.7%) were recorded under the source-defined category of other or self-arranged employment (Table 3).

**Table 3:** At-graduation destinations of the first graduating cohort (*n* = 28).

**Destination category**	Route or detail	*n*	%
Further study	Postgraduate entrance examination	4	14.3
	Recommendation-based admission	2	7.1
	Overseas postgraduate study	1	3.6
Civil-service or public-sector recruitment	–	3	10.7
State-owned enterprise employment	–	1	3.6
Other or self-arranged employment	–	17	60.7
Total		28	100

Categories are reported as recorded in the aggregate source. The “other or self-arranged employment” category was not further disaggregated.

The data therefore provide an early profile of graduate transition rather than a longitudinal employment outcome. The aggregate source did not distinguish programme-related employment within the broad other or self-arranged category and did not include six-month employment status, employment stability, employer feedback or workplace competence.

## 4 Discussion

### 4.1 Graduate identity and programme coherence

This longitudinal case study identified a consistent pattern across programme development: formal curriculum provision expanded more rapidly than the mechanisms needed to integrate, assess and review that provision as a coherent whole. The revised curriculum positioned management more explicitly as a principal disciplinary foundation while increasing biomedical, public health and digital health content. Teaching responsibilities were distributed across academic and practice settings, and the 40-week internship established a broad placement structure with common activity requirements. However, the five-stage analysis showed substantially less documentary continuity at the stages of practice, assessment and feedback than at the stages of stated intention and curricularisation. This pattern is consistent with the distinction between prescribed curriculum and its subsequent enactment and institutionalization described in curriculum implementation research (16).

These findings raise a more fundamental question than whether an HSM curriculum contains enough medical or management content: what kind of graduate is the programme intended to produce? In China, HSM is formally classified within Public Administration under the broader discipline of Management rather than within the medical disciplines (17). At the same time, HSM programmes are intended to prepare graduates for work in health services and organizations shaped by disease, clinical processes, population health and increasingly complex care needs (11–15). This creates a distinctive educational boundary. Students require sufficient clinical and public health literacy to understand the systems they may later analyse or manage, but such knowledge serves a different purpose from preparation for clinical practice. Conversely, management education risks losing disciplinary and professional relevance if it is taught as generic theory with little connection to healthcare organizations and service problems. The central curriculum issue is therefore not a fixed proportion of medical and management courses, but the function that each body of knowledge serves in developing an identifiable HSM graduate capability.

Clarifying this identity is increasingly important as health systems respond to population aging, chronic and complex care needs and greater integration across hospital, community, primary care and long-term care settings. WHO guidance on integrated care for older people similarly emphasizes coordinated, person-centered care across organizational and service boundaries (18). Such developments strengthen the rationale for professionals who understand health services while contributing primarily through management, coordination, analysis and improvement rather than clinical practice. From this perspective, hospital embedding is an educational resource rather than a professional identity in itself. Hospitals can provide access to clinical processes, organizational problems, data, practitioners and management settings, but the educational value of these resources depends on how they are organized around clearly defined graduate outcomes, progressive learning and interpretable assessment. The present case therefore suggests that graduate identity and occupational positioning provide a reference point for resource-to-capability conversion: a programme needs a clear account of what its graduates are expected to understand, analyse and manage before distributed institutional resources can be organized coherently around those purposes. This case-derived proposition provides the conceptual basis for the discussion that follows.

### 4.2 Disciplinary boundaries and knowledge integration

The curriculum changes observed in this case make the knowledge boundary of HSM particularly visible. The revised programme positioned management more explicitly as a principal disciplinary foundation while also expanding biomedical and health science content. At the same time, the documentary record did not show a clear longitudinal sequence through which these disciplinary components were progressively connected, applied and assessed. This pattern suggests that the central curriculum question is not simply how much medical or management content should be included, but what educational function each field should serve in the development of HSM graduates.

In China, HSM is formally classified within Public Administration under the broader discipline of Management rather than within the medical disciplines (17). This disciplinary position distinguishes health and clinical literacy within HSM from education intended to prepare students for licensed clinical practice (19). Medical and public health knowledge nevertheless remains important because management decisions in health services are shaped by disease burden, patient pathways, clinical workflows, quality and safety, prevention and population health needs. The purpose of such content is therefore not to reproduce a shortened medical curriculum, but to enable graduates to understand the organizations, processes and populations they may later analyse, coordinate or manage. This is consistent with broader calls for health professions education to align knowledge and learning with health system needs and intended capabilities (1, 2).

Management knowledge requires an equally deliberate form of contextualization. Retaining management as the disciplinary foundation of HSM does not imply transferring a generic management curriculum unchanged into a medical university. Concepts from organization, strategy, human resources, operations, quality improvement, finance, project management and data analysis become professionally relevant when they are applied to health service problems and organizational settings. Previous studies of HSM education in China have similarly identified disciplinary positioning, curriculum coherence and alignment with occupational needs as continuing challenges (13–15). From this perspective, the objective is not the parallel accumulation of medical and management courses, but purposeful integration around the kinds of problems graduates are expected to address. Constructive alignment and curriculum integration theory likewise emphasize connections among intended outcomes, learning experiences and assessment rather than the simple coexistence of disciplinary content (3, 20, 21).

A more coherent curriculum architecture would therefore proceed from occupational positioning to graduate capabilities and then to curriculum content. Programme-level capabilities could first define what graduates should be able to understand, analyse, coordinate and improve; medical, public health, management, research and digital content could then be mapped to those capabilities according to where they are introduced, reinforced, applied and assessed across the programme. The 2026 standards of the Commission on Accreditation of Healthcare Management Education similarly emphasize alignment among programme mission, competencies, curriculum and postgraduate outcomes (22). Although these standards were developed for graduate healthcare management education, they provide a useful comparative reference for considering programme-level alignment. Such an approach would shift curriculum review away from balancing disciplinary credit volumes toward clarifying the contribution of each component to graduate capability, while also providing a clearer foundation for later-stage differentiation according to occupational or further-study pathways.

### 4.3 Distributed expertise and collective ownership

The distributed teaching model observed in this case reflects both an opportunity and a coordination challenge. Course-level responsibilities were clearly allocated across university teaching units, affiliated hospitals and external contributors, but programme-level responsibility for curriculum sequencing, assessment and review was less explicit. The faculty-development documents also identified shortages of staff with directly relevant HSM backgrounds and limited dedicated staffing. These findings suggest that the central issue is not simply the number of teachers available to the programme, but how different forms of expertise are combined around a shared curriculum.

For an interdisciplinary HSM programme, disciplinary expertise is complementary rather than interchangeable. Medical and public health faculty can contribute knowledge of disease, care processes, patient pathways and health system context; management academics can provide conceptual and methodological foundations in organization, strategy, operations, human resources and related fields; hospital managers and external practitioners can bring current organizational problems and workplace experience into teaching. None of these contributions alone is sufficient to define the programme. The educational task is to connect them so that management concepts are applied to health service problems while health and clinical knowledge remains relevant to the graduate's managerial rather than clinical role. This is consistent with literature on distributed health professions education, where access to multiple settings and contributors requires deliberate coordination if it is to support a coherent programme (9, 10).

This perspective shifts the governance question away from whether teachers are full-time, part-time, academic or practitioner based. What matters is whether responsibility for individual courses is connected to collective responsibility for the graduate pathway. Curriculum governance therefore needs a programme-level structure with authority over intended outcomes, course boundaries, progression, assessment and periodic review, while allowing contributing units to retain responsibility for disciplinary teaching (23). In a hospital-embedded programme, such a structure could also support more deliberate collaboration between management academics, health science faculty and experienced healthcare managers through joint course planning, shared cases and co-developed practice tasks. Their contribution could then be examined through evidence on teaching practice, student experience and curriculum outcomes.

### 4.4 Common foundations and differentiated pathways

The curriculum findings raise the question of whether continued expansion of a single common curriculum is the best way to accommodate the breadth of HSM. In this case, graduation credits and required courses increased, while the longitudinal relationship among management, biomedical, public health and digital content remained less explicit. Programme objectives and placement arrangements also encompassed a wide range of potential employment settings, including hospitals, community services, public health organizations and health-related industries. Early graduate destinations were similarly diverse, although the available at-graduation data could not establish the degree of alignment between study and subsequent employment. These findings suggest that curriculum breadth may be better organized through a shared foundation combined with opportunities for greater depth in selected areas.

A common-core-and-pathway model offers one possible approach. The earlier years could establish shared capabilities in health and clinical literacy, public health, management, research and data use, communication, ethics and digital health. Curriculum mapping could identify where these capabilities are introduced, reinforced, applied and assessed, reducing reliance on repeated addition of stand-alone courses and strengthening connections among intended outcomes, learning experiences and assessment (3, 20, 21). Greater differentiation could then be introduced in the later stages through coordinated elective clusters, practice placements and applied projects. Possible pathways might include hospital management, health and chronic-care management, digital health and health information, public health and policy, or health-industry settings. The aim would be to allow greater depth without fragmenting the shared HSM foundation.

Pathway design should be informed by student aspirations, longitudinal graduate destinations, employer and placement-supervisor perspectives, and local workforce demand. Students oriented toward hospital management, for example, might pursue deeper learning in operations, quality, payment or workforce management, whereas those oriented toward digital health might focus more heavily on information systems, data use and digital governance. Different pathways could therefore support distinct occupational or postgraduate trajectories while remaining anchored to common programme-level capabilities and assessment expectations. Future evaluation could examine whether this form of later-stage differentiation improves perceived relevance, employment alignment and competency development while preserving programme coherence. In this way, differentiated pathways would become part of a broader curriculum architecture rather than a set of loosely connected specializations.

### 4.5 Diverse practice and comparable assessment

The practice findings reveal an important distinction between diversity of learning experience and comparability of performance evidence. The 40-week internship included hospital, public health, community and industry settings, with common activity requirements, supervision and documentation. However, the absence of shared performance anchors and weighting across sites limited the extent to which evidence from different placements could be interpreted against common expectations. This issue becomes particularly important if later-stage differentiation is introduced. Students may undertake different tasks and develop greater depth in different settings, but their performance can still be judged against shared HSM capabilities. Workplace-based and programmatic assessment support this principle by emphasizing multiple observations and forms of evidence over time rather than isolated activity counts or single judgements (7, 24, 25).

Comparable assessment therefore requires more than standardizing placement activities. Documented assessment sources in this case included course assessments, internship records, departmental evaluations, reflections and final examinations, but these sources were not consistently connected through a programme-level account of capability progression. A shared competency framework and developmental rubrics could provide common reference points while allowing learning experiences to remain context specific. Students in hospital management, community health, digital health or industry-oriented pathways could encounter different problems and settings, yet still generate evidence relating to common capabilities such as analyzing service problems, using data, coordinating stakeholders, developing interventions and evaluating implementation. Such an approach would preserve diversity in learning while making progression and performance more interpretable across the programme.

Assessment also becomes more valuable when it contributes to programme learning. Teaching evaluations, inspection instruments and graduate-transition data provided potential sources of programme-level evidence, but the five-stage analysis identified little documentary evidence that these sources were systematically connected to subsequent curriculum decisions and reassessment. A stronger feedback cycle would link evidence collection, synthesis, curriculum decisions, implementation and review (26). Longitudinal graduate follow-up, together with student, placement-supervisor and employer perspectives, could further show whether the common core, differentiated pathways and assessment arrangements remain aligned with changing professional and workforce needs (27–29). Practice and assessment would then serve not only to document student progression, but also to provide evidence for continuing curriculum development.

### 4.6 Distributed resources and coherent capability

The findings across curriculum architecture, distributed teaching, practice education, assessment and quality assurance can be brought together through a provisional resource-to-capability conversion framework (Figure 4). Graduate identity and occupational positioning provide the reference point for organizing the framework, because the value of educational resources depends on the capabilities a programme intends its graduates to develop. In a hospital-embedded setting, these resources may include disciplinary expertise, healthcare organizations, management problems, operational data, practice settings and professional networks. Their presence expands educational opportunity, but coherence depends on how they are organized across the graduate pathway.

**Figure 4:**
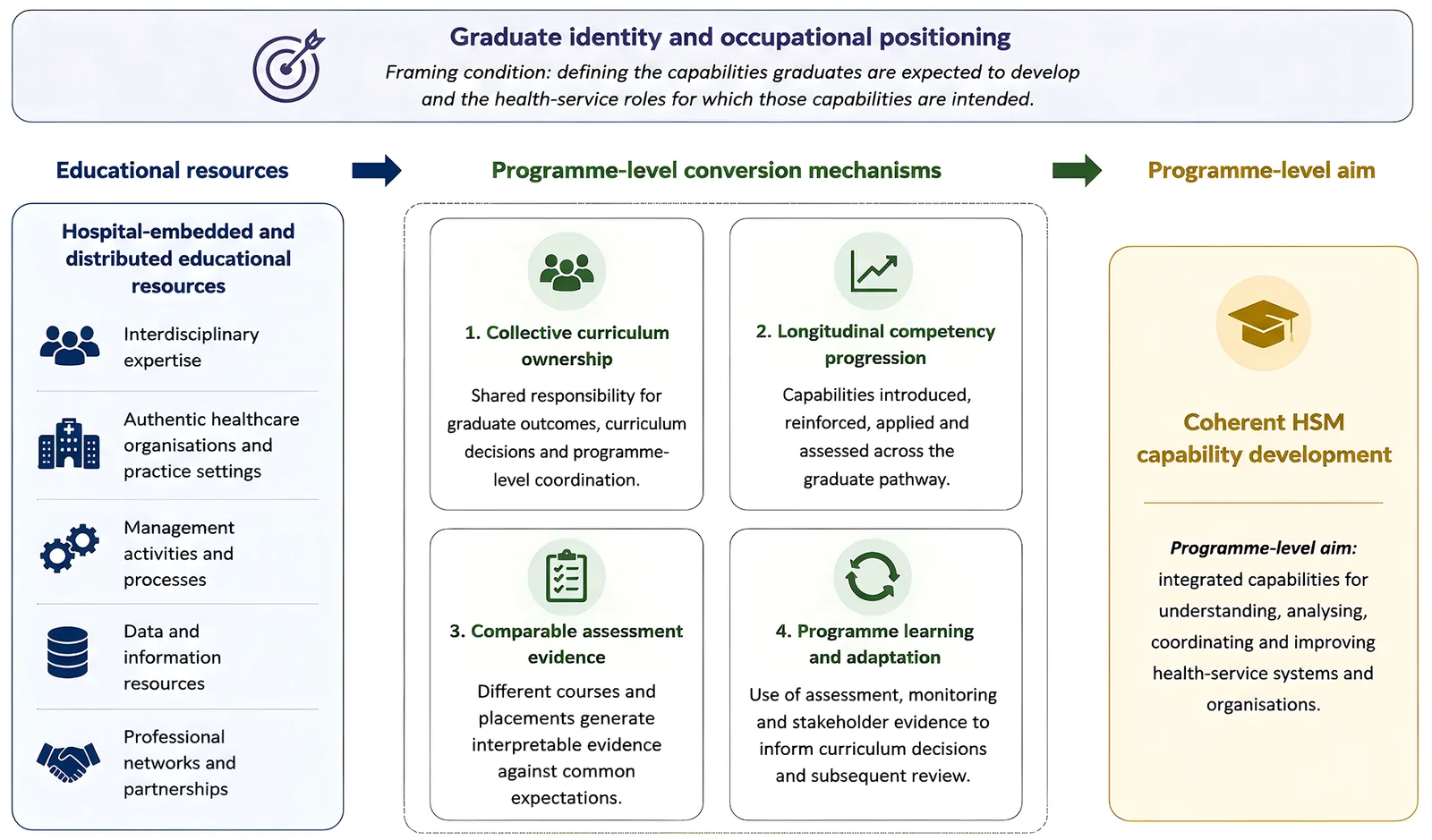
Provisional resource-to-capability conversion framework derived from the present case. Graduate identity and occupational positioning provide the framing condition for organizing hospital-embedded and distributed educational resources. Four programme-level mechanisms, namely collective curriculum ownership, longitudinal competency progression, comparable assessment evidence, and programme learning and adaptation, represent the processes through which these resources may contribute to coherent HSM capability development. Arrows indicate proposed conceptual relationships among the framework components rather than tested causal effects.

Four programme-level mechanisms were particularly salient in this case. Collective curriculum ownership connects contributions from different academic units, hospitals and external practitioners to shared programme outcomes and programme-level responsibility (23). Longitudinal competency progression links curriculum content across stages of introduction, reinforcement, application and assessment (3, 20, 21). Comparable assessment evidence allows different courses and practice settings to contribute interpretable evidence against common expectations without requiring identical learning experiences (7, 24, 25). Programme learning and adaptation connect assessment, monitoring and stakeholder evidence with curriculum review and subsequent improvement (26). Together, these mechanisms address the main discontinuities identified between curriculum breadth, distributed responsibility, diverse practice and programme-level coherence.

The framework therefore shifts attention from the quantity of available resources to the educational and organizational processes through which those resources are made coherent. It also accommodates differentiated pathways, because students may pursue different elective, practice or project experiences while remaining connected to shared HSM capabilities and assessment expectations. Resource-to-capability conversion, in this sense, describes the work required to connect institutional resources with a coherent graduate pathway rather than a fixed developmental sequence. The framework is best understood as a case-derived interpretive lens that generates propositions for further examination across different programme settings.

### 4.7 Evidence boundaries and future inquiry

This study followed the programme from approval to the graduation of its first cohort and drew on successive curriculum, teaching, internship, assessment and quality assurance documents. Prespecified indicator definitions, traceable documentary evidence, independent rescoring of all 33 indicators and high inter-rater agreement strengthened the transparency of the analysis. The five-stage framework further allowed programme development to be examined across stated intentions, curriculum provision, practice, assessment and feedback, making document analysis particularly useful for tracing programme architecture and institutional change over time (30).

The analysis addresses the documented curriculum rather than the full educational experience. Institutional documents can establish what was formally planned, assigned, required or recorded, but they cannot determine how consistently these arrangements were enacted, how students and teachers experienced them or whether graduates attained the intended capabilities. Documentary absence therefore does not establish non-implementation, just as documentary presence does not establish educational effectiveness. The first author's involvement in programme development provided knowledge of document provenance and context, while prespecified criteria, evidence traceability and independent review helped distinguish contextual knowledge from documentary evidence (31). The single-case design also limits wider generalization, and the graduate data provide only an at-graduation transition profile rather than evidence of employment stability, occupational alignment or workplace competence (32).

Future research should extend this documentary account through evidence from students, teachers, placement supervisors, graduates and employers, together with observation of teaching and practice where feasible. Longitudinal graduate follow-up could examine employment pathways, programme relevance and employer perspectives (27–29). Particular attention should be given to how students understand HSM professional identity in a medical university, how different curriculum components contribute to that identity and whether differentiated pathways align with career intentions while preserving common programme-level capabilities. Multi-site studies could further examine whether the relationships among graduate identity, curriculum ownership, competency progression, assessment comparability and programme learning are evident across programmes with different institutional arrangements.

## 5 Conclusion

This longitudinal case study suggests that the central challenge for a hospital-embedded undergraduate HSM programme is not simply how much medical or management content it contains, nor how many institutional resources it can mobilize, but whether those elements are organized around a coherent graduate identity. The documentary record showed substantial curriculum breadth, distributed teaching arrangements and diverse practice opportunities, while longitudinal integration, comparable assessment and programme-level feedback remained less fully documented. The case therefore points to the importance of collective curriculum ownership, progressive capability development, interpretable assessment and programme learning in connecting distributed resources to coherent education. For HSM in a medical university, the task is neither to approximate clinical training nor to reproduce generic management education, but to define a distinctive professional space in which health and clinical literacy give context to management capability. The provisional framework proposed here offers one way to examine that space. Ultimately, the future of HSM may depend less on choosing between medicine and management than on whether it can establish a credible identity at their intersection.

## Data Availability

The original contributions presented in the study are included in the article/Supplementary material, further inquiries can be directed to the corresponding authors.

## Supplementary material

Supplementary Table S1 Inventory of the 19 cataloged records, including one duplicate record, eligibility decisions and analytical roles for the 18 unique records, of which 15 formed the empirical corpus and three were retained for non-empirical or contextual purposes.

Supplementary Table S2 Complete definitions of the 33 indicators, documentary evidence sources, scoring criteria, source locations, independent first- and second-reviewer ratings and final consensus scores.

Supplementary Table S3 Indicator-level evidence matrix across the five analytical stages (stated, curricularised, practiced, assessed and fed back), including not-applicable designations.
