# Supplementary material for: Curriculum evolution and documentary evidence of competency alignment in a hospital-embedded undergraduate health services and management programme in China: a longitudinal case study
Source: Front Public Health. 2026 Sep 24;14:1956968. doi: 10.3389/fpubh.2026.1956968 (PMC13646136; doi:10.3389/fpubh.2026.1956968)
Supplement: Supplementary file 1 [file Data_Sheet_1.docx]

**Supplementary Tables**

**Supplementary Table S1. Inventory, eligibility and analytical role of the identified documents**

| **ID** | **Document** | **Version/date** | **Type/authority** | **Eligibility decision** | **Analytical role** | **Key limitation** |
| --- | --- | --- | --- | --- | --- | --- |
| D01 | Original four-year undergraduate HSM training plan (version 0523) | Original plan; 0523 version | Formal training plan | Included | Baseline curriculum and programme requirements | Internal inconsistencies in total contact hours and practice-credit definitions were retained. |
| D02 | Revised four-year undergraduate HSM training plan (version 1009) | Revised plan; 1009 version | Revised training plan | Included | Longitudinal curriculum comparison and revised positioning | The plan refers to three attachments, but revised-version attachments were not identified. |
| D03 | Required-course progression table for the original plan | Attachment 1; 0523 version | Curriculum attachment | Included | Original required-course details | The filename links it to the original, not the revised, plan. |
| D04 | Elective-course plan for the original programme | Attachment 3; 0523 version | Curriculum attachment | Included | Original elective-course provision | Available electives do not establish actual enrolment or competence attainment. |
| D05 | Course-to-programme-requirement mapping matrix | Attachment 4; original-plan matrix | Curriculum map | Included | Original course-requirement mapping | The matrix lacks mapping intensity, progression and assessment detail; its relationship to the revised plan is unconfirmed. |
| D06 | Application for establishment of HSM teaching units | Version 6.7; year not stated | Organisation/planning document | Included | Teaching-unit structure and course ownership | Proposed units and leads were not treated as proof of sustained operation. |
| D07 | Responsibilities of teachers in the HSM teaching unit | Undated | Role description | Included | Teacher and course responsibilities | Formal duties do not establish completed performance. |
| D08 | Overall faculty-development plan for the HSM teaching unit | Undated planning document | Faculty plan | Included | Faculty-capacity diagnosis and development goals | Leadership information is outdated; targets and measures are not achieved outcomes. |
| D09 | Teacher forum opinion-summary form | Blank template; undated | Feedback instrument | Included | Teacher-feedback infrastructure | Shows that an instrument exists, not that feedback was collected or acted upon. |
| D10 | HSM programme reform report | Internal reform period; year not stated | Internal reform report | Included | Problem diagnosis and proposed reform | Recommendations and proposed measures were not treated as completed implementation. |
| D11 | Graduation internship arrangement for the 2022 cohort | 2025-2026; dated 9 July 2025 | Implementation schedule | Included | First-cohort 40-week internship structure and requirements | Requirements do not demonstrate completion quality. |
| D12 | Detailed HSM internship handbook | First-cohort internship period | Practice record and assessment tool | Included | Setting-specific tasks, records and assessment | No behaviourally anchored rubric or common weighting system was provided. |
| D13 | Teaching-quality evaluation form for professional theory courses | Blank template; undated | Quality instrument | Included | Teacher self-evaluation infrastructure | Assesses teaching process, not student competence; no completed scores were supplied. |
| D14 | Mid-semester teaching inspection form for teacher self-review | Blank/semi-structured template; undated | Quality instrument | Included | Mid-semester process monitoring | Shows monitoring infrastructure only; completed records and follow-up action were unavailable. |
| D15 | Aggregate progression and employment summary for the first cohort | At graduation, June 2026 | Aggregate outcome image | Included | Early graduate-transition information | Not a six-month employment rate or evidence of job relevance, stability or competence; the other/self-arranged category is broad. |
| C01 | Proposal for a graduate discipline in hospital management | Version 6; 28 July 2026 | Contextual document | Context only | Subsequent institutional trajectory | Post-dates undergraduate curriculum revision and was not coded as undergraduate implementation evidence. |
| R01 | Digital upgrading of a health-services programme cluster under New Medical Education | External document; undated | External reference | Sensitising reference | Digital-reform concepts | Concerns another institution and vocational education; not empirical evidence for the case programme. |
| X01 | Undergraduate HSM curriculum from another university | External curriculum PDF | External curriculum | Excluded | No empirical role | Different institution and organisational context. |
| DUP01 | Duplicate copy of the first-cohort internship arrangement | Duplicate of D11 | Duplicate record | Duplicate removed | No separate analytical role | Content and file hash matched D11; removed before eligibility assessment. |

**Data provenanc**e: Tables S2 and S3 were reconstructed directly from the locked first-round coding matrix and the completed blinded second-round review workbook. The two recorded disagreements were indicators 1.2 and 3.5; both were resolved conservatively at score 1 in the final consensus dataset.

**Note:**  Nineteen records were initially identified. Removal of DUP01 left 18 unique materials for eligibility assessment: 15 programme-specific empirical materials (D01-D15), one contextual document (C01), one sensitising reference (R01) and one excluded external curriculum (X01). Institution-neutral English titles are used for manuscript presentation.

**Supplementary Table S2. Indicator definitions, documentary evidence, independent ratings and final consensus scores for the 33 indicators**

| **Code** | **Indicator definition** | **Documentary evidence** | **Sources and location** | **First-round rating** | **Independent rating** | **Final consensus** |
| --- | --- | --- | --- | --- | --- | --- |
| **Programme objectives and disciplinary positioning** | | | | | | |
| 1.1 | Management as an explicit disciplinary foundation. Management is explicitly positioned as a principal discipline and supported by formal management-course provision. | The original plan foregrounded medical and public-health fields; the revised plan explicitly added management as a main discipline and listed Management Principles, Management Communication and Hospital Management. | D01, section 5; D02, sections 5 and 8 | 2 | 2 | 2 |
| 1.2 | Systematic integration of biomedical and public-health knowledge. Biomedical and public-health content is connected through integrated curriculum design or interdisciplinary learning mechanisms rather than only listed in parallel. | Both plans require biomedical, clinical, epidemiological and public-health knowledge, and the revised plan adds several biomedical courses; however, the evidence mainly comprises parallel content and course lists rather than an explicit integration mechanism. | D01 and D02, learning requirements, curriculum and practice sections | 2 | 1 | 1 |
| 1.3 | Alignment of programme objectives with occupational and industry needs. Programme objectives identify specific employment settings or roles and are supported by corresponding practice opportunities. | The revised objectives identify hospitals, community health centres, health-management enterprises, insurers and health-administration bodies, while the first-cohort internship covers corresponding hospital, CDC, community, elder-care, insurance and health-communication settings. | D02, objectives; D11, placement schedule; D12, placement scope | 2 | 2 | 2 |
| 1.4 | Complete objectives for digital health, artificial intelligence and data capability. Digital, AI and data capabilities are stated as graduate outcomes and supported through curriculum, applied learning and assessment. | The revised plan repeatedly states digital thinking, AI, data analysis and health-information capability and includes Medical AI Foundations and Health Information Management; however, AI and industry-integration modules are recorded as containing zero courses and no dedicated capability assessment was identified. | D02, introduction, objectives and curriculum; D11, clinical/administrative rotation | 1 | 1 | 1 |
| **Curriculum architecture and progression** | | | | | | |
| 2.1 | Explicit course-category, credit and contact-hour structure. Course categories, credits, contact hours, practice proportions and graduation requirements are formally specified. | The original plan specifies 165 credits and 47 required courses. The revised plan specifies 184.5 credits and categories for general education, professional education, distinctive development and practice, with contact hours and practice proportions. | D01-D04, credit requirements, curriculum and attachments | 2 | 2 | 2 |
| 2.2 | Traceable course-to-graduation-requirement mapping. Courses are mapped to programme requirements through a current, version-matched and auditable curriculum map. | A course-to-requirement matrix exists for the original plan. The revised plan refers to a matrix in an attachment, but no version explicitly corresponding to the revised plan was identified; the original matrix also lacks progression and assessment levels. | D05, mapping matrix; D02, section 10 | 1 | 1 | 1 |
| 2.3 | Semester-by-semester progression of capability development. The curriculum makes explicit how capabilities are introduced, reinforced, practised and assessed across semesters. | Required and elective courses are arranged by semester, with foundational courses earlier and professional courses later, but no introduced-reinforced-practised-assessed coding or prerequisite map was identified. | D03-D05, semester schedules and mapping matrix | 1 | 1 | 1 |
| 2.4 | Breadth and depth of the management curriculum. Management content has sufficient breadth, required status, depth and internal consistency to support the programme's disciplinary identity. | Management Principles, Management Communication and Hospital Management are present, while Organisational Behaviour, Human Resource Management and Health Project Management are electives; some core management content is optional and course lists are internally inconsistent. | D01, D02 and D04, core and elective course lists | 1 | 1 | 1 |
| 2.5 | Systematic review of curriculum expansion, duplication and trade-offs. Curriculum review explicitly examines workload, redundancy, additions, deletion and opportunity costs, with decisions traceable to implementation. | The reform report proposes compressing overview medical courses and removing overlapping management content, yet total credits increase from 165 to 184.5 and several biomedical courses are added. | D10, curriculum adjustment; D01-D02 comparison | 1 | 1 | 1 |
| **Distributed governance and faculty capacity** | | | | | | |
| 3.1 | Clear course ownership and accountable roles. Each course or teaching function has an identifiable responsible unit and documented faculty responsibilities. | The teaching-unit document allocates courses to schools, affiliated hospitals and proposed teaching groups and identifies responsible persons; teacher responsibilities cover syllabi, implementation plans, teaching materials and item banks. | D06, teaching-unit application; D07, teacher responsibilities | 2 | 2 | 2 |
| 3.2 | Operational cross-unit curriculum coordination. A defined mechanism coordinates planning, sequencing, assessment and review across distributed teaching units and is evidenced in operation. | Documents propose multi-unit delivery, a reform working group and collective lesson preparation, but no curriculum-committee minutes, cross-unit coordination records or consolidated reporting mechanism were identified. | D06 and D10, teaching-unit structure, collective preparation and reform group | 1 | 1 | 1 |
| 3.3 | Sufficient faculty with relevant HSM backgrounds. The programme has adequate faculty capacity with directly relevant HSM or management expertise. | The faculty-development plan explicitly states that faculty with relevant HSM backgrounds are few, dedicated staffing is insufficient and senior/highly qualified faculty are limited. | D08, situation analysis | 1 | 1 | 1 |
| 3.4 | Targeted faculty-development mechanism. Identified faculty gaps are addressed through implemented recruitment, training, mentoring, development and evaluation arrangements. | Plans specify recruitment, targeted training, academic exchange, evaluation and qualification targets, plus collective lesson preparation; participation numbers, frequency and outcomes are not reported. | D08 and D10, implementation and faculty-development sections | 1 | 1 | 1 |
| 3.5 | Effective engagement of hospital managers and external practice experts. Hospital managers and external experts participate through defined, implemented and sustained teaching or curriculum roles. | Documents state that hospital professors, leaders and managers are proposed as course or teaching-group leads and recommend industry or management experts for teaching; wording is prospective and sustained participation records are unavailable. | D06 and D10, course-lead arrangements and faculty adjustment | 2 | 1 | 1 |
| **Practice education and cross-setting comparability** | | | | | | |
| 4.1 | Diverse and authentic practice settings. The documented placement structure includes hospital, public-health, community and industry settings relevant to HSM practice. | The 40-week internship covers hospital health-management centres, clinical and administrative departments, the provincial CDC, community health centres, health communication, fitness-health integration, elder care and insurance. | D11 and D12, placement arrangement and handbook | 2 | 2 | 2 |
| 4.2 | Common core tasks and minimum requirements across settings. Programme documents specify shared minimum activities or outputs for all students across the internship. | Common requirements include at least 200 health-management records, 30 health-education activities and 100 chronic-disease follow-ups, plus a learning reflection and internship report. | D11, internship requirements and assessment | 2 | 2 | 2 |
| 4.3 | Setting-specific learning objectives and tasks. Internship documents specify objectives and tasks for different practice settings according to their service and management contexts. | The handbook lists distinct tasks for hospital health-management centres, community care, health-management companies, elder-care institutions, insurers and health-administration or public-service organisations. | D12, setting-specific internship records | 2 | 2 | 2 |
| 4.4 | Explicit supervision, recording and sign-off arrangements. Supervisory responsibility, practice records, evaluation and sign-off procedures are formally defined. | Supervisors, department heads or responsible units must complete and sign records; placement sites submit original assessment materials and results, while the handbook records weekly activity, performance, skills and overall comments. | D11-D12, handbook instructions, logs and assessment | 2 | 2 | 2 |
| 4.5 | Comparability of assessment standards and results across sites. Common criteria, anchors and weighting enable performance to be compared across placement sites. | All sites assess students, but the internship arrangement permits assessment to be organised according to each site's circumstances; the handbook lacks common behaviourally anchored standards and weights. | D11-D12, assessment sections and registration form | 1 | 1 | 1 |
| **Assessment architecture** | | | | | | |
| 5.1 | Combination of formative and summative assessment. Assessment includes defined formative and summative components across courses and practice learning. | The plans define formative assessment through online self-tests, in-class tests, assignments, laboratory reports and case records, and summative assessment through final examinations and skills tests; internship assessment adds continuous performance and a graduation written examination. | D01, D02 and D11, assessment and graduation examination | 2 | 2 | 2 |
| 5.2 | Behaviourally anchored assessment of management competence. Management competencies are decomposed into observable behaviours and performance levels within formal rubrics. | The handbook assesses performance, attitude, skills and attendance and refers to management and workplace adaptation, but does not translate communication, coordination, data use, project management and problem-solving into observable performance levels. | D12 and D11, assessment form and internship objectives | 1 | 1 | 1 |
| 5.3 | Multiple-source and longitudinal assessment evidence. Programme documents specify assessment evidence from multiple assessors, records, tasks and time points rather than reliance on a single examination. | Evidence includes task volumes, stage logs, reflections, self-summary, supervisor evaluation, an internship report, departmental continuous assessment and the graduation written examination. | D11-D12, requirements, logs, site comments and graduation examination | 2 | 2 | 2 |
| 5.4 | A common core assessment tool across placement sites. A shared assessment tool with common anchors, weights and assessor guidance is used across sites. | A common recording form exists, but sites may organise assessment differently and no shared scoring anchors, weights or assessor training are specified. | D11-D12, internship assessment | 1 | 1 | 1 |
| 5.5 | Explicit progression and graduation decision rules. Assessment results are linked to transparent progression, eligibility, weighting, pass and graduation rules. | Failure of continuous internship assessment prevents entry to the graduation examination, and the final result combines the written examination with an average score; the composition, site weights and pass standards are unclear. | D11, assessment and graduation examination | 1 | 1 | 1 |
| **Digital-health competency maturity** | | | | | | |
| 6.1 | Dedicated digital-health or AI courses. Digital-health and AI capabilities are supported by identifiable formal courses within a coherent curriculum sequence. | The revised plan makes Medical AI Foundations required and retains Health Information Management; the original electives include Python, AI and big-data analysis, but the revised course-category table records zero courses in the AI module. | D02-D04, foundation, core and elective course lists | 1 | 1 | 1 |
| 6.2 | Authentic or applied digital learning tasks. Students undertake defined data, information-system, AI or digital-health tasks with specified outputs. | The revised plan refers to data analysis, electronic health records and digital health-management practice, and the internship includes a smart-hospital department; no syllabus, defined project task or output example was identified. | D02, programme features; D11, smart-hospital rotation | 1 | 1 | 1 |
| 6.3 | Explicit assessment of digital-health or AI capability. Formal assessment measures data use, information-system application, AI evaluation or digital governance capability. | Digital and AI capabilities are stated, but existing assessment tools contain no dedicated items for data analysis, health-information-system use, AI evaluation or digital governance. | D02, D11 and D12, objectives, placement and assessment records | 0 | 0 | 0 |
| 6.4 | Coverage of privacy, security, ethics and AI governance. Curriculum and assessment explicitly address health-data privacy, security, algorithmic bias, accountability, transparency and governance. | Medical ethics and health law are required and information security appears in the original elective list, but health-data privacy, algorithmic bias, accountability, transparency and AI governance are not explicitly specified. | D02 and D04, ethics, health law and information-security elective | 1 | 1 | 1 |
| **Quality assurance and feedback loops** | | | | | | |
| 7.1 | Formal teaching-quality monitoring instruments. Programme documents include tools designed to monitor teaching objectives, methods, learning activities, assessment and delivery. | A 30-item professional-theory teaching evaluation form and a mid-semester inspection form define monitoring items for objectives, methods, assignments, attendance, continuous assessment and teaching reform; completed monitoring records were not available. | D13-D14, teacher self-evaluation and mid-semester inspection | 2 | 2 | 2 |
| 7.2 | Systematic collection of teacher and student feedback. Teacher and student feedback is collected, analysed and reported through completed programme processes. | Teacher-forum, teacher self-evaluation and mid-semester inspection tools exist and the reform report proposes teacher and student feedback, but no completed forum record, student evaluation dataset or feedback analysis was identified. | D09, D10 and D13-D14, feedback and monitoring instruments | 1 | 1 | 1 |
| 7.3 | Employer and industry participation in curriculum decisions and quality improvement. Employers and industry partners contribute evidence to curriculum review, decisions and improvement. | The reform report proposes industry experts in the reform group, agreements with placement bases and an employment pipeline, and the internship includes four industry-placement types; employer participation in curriculum decisions or feedback results is not documented. | D10-D11, reform group, industry cooperation and placements | 1 | 1 | 1 |
| 7.4 | Traceable graduate destinations and job-relevance follow-up. Graduate destinations, job roles and programme relevance are followed beyond a broad at-graduation status summary. | For the 28 graduates, the at-graduation summary records further study 7 (25.0%), public-service recruitment 3 (10.7%), state-owned enterprise employment 1 (3.6%) and other/self-arranged employment 17 (60.7%); six-month status, job relevance, stability and employer feedback are unavailable. | D15, complete aggregate image | 1 | 1 | 1 |
| 7.5 | Use of feedback for curriculum revision and reassessment. A complete feedback cycle links evidence collection, decision-making, implementation and subsequent reassessment. | The reform report proposes curriculum evaluation, needs analysis, periodic outcome review, stakeholder feedback and adjustment, but no complete feedback-decision-implementation-reassessment record was identified. | D10, implementation steps | 1 | 1 | 1 |

**Note: (1)** Ratings used a three-category documentary-evidence scale: 0 = no relevant evidence identified; 1 = relevant evidence present but partial, planned, inconsistent or incomplete relative to the operational definition; 2 = explicit documentary evidence for the elements specified in the operational definition. Where an indicator required implementation, progression, cross-setting consistency, assessment or feedback, the presence of a related course, activity, form or statement of intent alone was insufficient for a score of 2. Ratings describe documentary representation rather than educational quality, implementation fidelity or competence attainment; absence of documentary evidence denotes absence from the available corpus rather than evidence that an activity did not occur in practice. The two highlighted rows (1.2 and 3.5) were the only disagreements; each was scored 2 in the first round and 1 in the independent review and was assigned a final consensus score of 1 after source re-examination. (2) Exact agreement was 31/33 (93.9%); unweighted Cohen's kappa = 0.879, linear-weighted kappa = 0.884 and quadratic-weighted kappa = 0.893. Final distribution: score 2, n=11; score 1, n=21; score 0, n=1.

**Supplementary Table S3. Evidence across the stated, curricularised, practised, assessed and fed-back stages**

| **Code** | **Indicator** | **Stated** | **Curricularised** | **Practised** | **Assessed** | **Fed back** | **Documentary anchor** |
| --- | --- | --- | --- | --- | --- | --- | --- |
| **Programme objectives and disciplinary positioning** | | | | | | | |
| 1.1 | Management as an explicit disciplinary foundation | Yes | Yes | Yes | No | No | D01, section 5; D02, sections 5 and 8 |
| 1.2 | Systematic integration of biomedical and public-health knowledge | Yes | Yes | No | No | No | D01 and D02, learning requirements, curriculum and practice sections |
| 1.3 | Alignment of programme objectives with occupational and industry needs | Yes | Yes | Yes | No | No | D02, objectives; D11, placement schedule; D12, placement scope |
| 1.4 | Complete objectives for digital health, artificial intelligence and data capability | Yes | Yes | Yes | No | No | D02, introduction, objectives and curriculum; D11, clinical/administrative rotation |
| **Curriculum architecture and progression** | | | | | | | |
| 2.1 | Explicit course-category, credit and contact-hour structure | N/A | Yes | N/A | N/A | No | D01-D04, credit requirements, curriculum and attachments |
| 2.2 | Traceable course-to-graduation-requirement mapping | Yes | Yes | No | No | No | D05, mapping matrix; D02, section 10 |
| 2.3 | Semester-by-semester progression of capability development | Yes | Yes | No | No | No | D03-D05, semester schedules and mapping matrix |
| 2.4 | Breadth and depth of the management curriculum | Yes | Yes | Yes | No | No | D01, D02 and D04, core and elective course lists |
| 2.5 | Systematic review of curriculum expansion, duplication and trade-offs | Yes | No | No | No | No | D10, curriculum adjustment; D01-D02 comparison |
| **Distributed governance and faculty capacity** | | | | | | | |
| 3.1 | Clear course ownership and accountable roles | N/A | Yes | Yes | No | No | D06, teaching-unit application; D07, teacher responsibilities |
| 3.2 | Operational cross-unit curriculum coordination | N/A | Yes | No | No | No | D06 and D10, teaching-unit structure, collective preparation and reform group |
| 3.3 | Sufficient faculty with relevant HSM backgrounds | N/A | Yes | No | No | No | D08, situation analysis |
| 3.4 | Targeted faculty-development mechanism | N/A | Yes | No | No | No | D08 and D10, implementation and faculty-development sections |
| 3.5 | Effective engagement of hospital managers and external practice experts | N/A | Yes | No | No | No | D06 and D10, course-lead arrangements and faculty adjustment |
| **Practice education and cross-setting comparability** | | | | | | | |
| 4.1 | Diverse and authentic practice settings | Yes | Yes | Yes | Yes | No | D11 and D12, placement arrangement and handbook |
| 4.2 | Common core tasks and minimum requirements across settings | Yes | Yes | Yes | Yes | No | D11, internship requirements and assessment |
| 4.3 | Setting-specific learning objectives and tasks | Yes | Yes | Yes | Yes | No | D12, setting-specific internship records |
| 4.4 | Explicit supervision, recording and sign-off arrangements | N/A | Yes | Yes | Yes | No | D11-D12, handbook instructions, logs and assessment |
| 4.5 | Comparability of assessment standards and results across sites | N/A | Yes | No | No | No | D11-D12, assessment sections and registration form |
| **Assessment architecture** | | | | | | | |
| 5.1 | Combination of formative and summative assessment | Yes | Yes | Yes | Yes | No | D01, D02 and D11, assessment and graduation examination |
| 5.2 | Behaviourally anchored assessment of management competence | Yes | Yes | Yes | No | No | D12 and D11, assessment form and internship objectives |
| 5.3 | Multiple-source and longitudinal assessment evidence | Yes | Yes | Yes | Yes | No | D11-D12, requirements, logs, site comments and graduation examination |
| 5.4 | A common core assessment tool across placement sites | N/A | Yes | No | No | No | D11-D12, internship assessment |
| 5.5 | Explicit progression and graduation decision rules | N/A | Yes | Yes | Yes | No | D11, assessment and graduation examination |
| **Digital-health competency maturity** | | | | | | | |
| 6.1 | Dedicated digital-health or AI courses | Yes | Yes | No | No | No | D02-D04, foundation, core and elective course lists |
| 6.2 | Authentic or applied digital learning tasks | Yes | Yes | Yes | No | No | D02, programme features; D11, smart-hospital rotation |
| 6.3 | Explicit assessment of digital-health or AI capability | No | No | No | No | No | D02, D11 and D12, objectives, placement and assessment records |
| 6.4 | Coverage of privacy, security, ethics and AI governance | Yes | Yes | No | No | No | D02 and D04, ethics, health law and information-security elective |
| **Quality assurance and feedback loops** | | | | | | | |
| 7.1 | Formal teaching-quality monitoring instruments | N/A | Yes | No | No | No | D13-D14, teacher self-evaluation and mid-semester inspection |
| 7.2 | Systematic collection of teacher and student feedback | N/A | Yes | No | No | No | D09, D10 and D13-D14, feedback and monitoring instruments |
| 7.3 | Employer and industry participation in curriculum decisions and quality improvement | Yes | Yes | No | No | No | D10-D11, reform group, industry cooperation and placements |
| 7.4 | Traceable graduate destinations and job-relevance follow-up | N/A | Yes | No | Yes | No | D15, complete aggregate image |
| 7.5 | Use of feedback for curriculum revision and reassessment | N/A | Yes | No | No | No | D10, implementation steps |

**Note:** Yes = documentary evidence relevant to that stage was identified; No = no such evidence was identified; N/A = the stage was not conceptually applicable. A Yes at one stage does not mean that the full indicator was satisfied. Stated indicates explicit recognition in objectives or plans; curricularised indicates translation into a course, practice arrangement, responsibility structure or formal instrument; practised indicates documentary evidence of a defined opportunity or implemented activity relevant to the indicator rather than direct observation of educational practice; assessed indicates a formal assessment, record, criterion, rubric, monitoring procedure or decision mechanism that addresses the indicator; fed back requires evidence that assessment or monitoring findings were subsequently used in programme decision-making, curriculum revision or improvement. The stage profile identifies discontinuities and is interpreted alongside the overall 0-2 rating; documentary presence does not establish implementation quality, learner experience or competence attainment.
